# Impacts of Heat Stress around Flowering on Growth and Development Dynamic of Maize (*Zea mays* L.) Ear and Yield Formation

**DOI:** 10.3390/plants11243515

**Published:** 2022-12-14

**Authors:** Na Wang, Qi Liu, Bo Ming, Wenxin Shang, Xuefeng Zhao, Xuqing Wang, Jing Wang, Junlong Zhang, Zhongkui Luo, Yong Liao

**Affiliations:** 1 Crop Research Institute, Shandong Academy of Agricultural Sciences, Jinan 250100, China; 2 College of Geography and Environment, Shandong Normal University, Jinan 250358, China; 3 Institute of Crop Science, Chinese Academy of Agricultural Sciences/Key Laboratory of Crop Physiology and Ecology, Ministry of Agriculture and Rural Affairs, Beijing 100081, China; 4 Collage of Agriculture, Qingdao Agricultural University, Qingdao 266109, China; 5 College of Resources and Environmental Sciences, China Agricultural University, Beijing 100193, China; 6 College of Environmental and Resource Sciences, Zhejiang University, Hangzhou 310058, China; 7 Agricultural Technology Extension Central Station of Jining, Jining 272000, China

**Keywords:** high temperature, ear traits, logistic model, heat-tolerant, kernel number per ear, 1000-grain weight

## Abstract

Heat stress around flowering is harmful to maize growth and yield. Ear traits are closely related to yield; however, the effects of heat stress before and after flowering on ear development and yield traits remain unclear for different heat-tolerant cultivars. In this study, field experiments were conducted in 2020 and 2021, including (i) three sowing dates, (ii) three temperature regimes: control (CK), heated before silking (V9-R1, TBS) and heated after silking (R1-R1 + 15 d, TAS), and (iii) two hybrids (ZD958: heat-tolerant; DH605: heat-sensitive). The results showed that heating had negative effects on all surveyed ear and yield traits except for increased ear length under TBS. The negative effects were larger (i) for TAS than for TBS, (ii) for DH605 than for ZD958, and (iii) for kernel number per plant (KNP) than for kernel weight (KW). The decreased ear traits were a result of a decreased growth rate during rapid ear growth periods. Floret pollination failure and kernel abortion were the main reasons for the decrease in KNP, mainly depending on the daily maximum temperature during V15-R1 + 7 d. The strong linear relationships between ear and yield traits suggested that ear traits could be used as important indicators for breeding heat-resistant varieties in the future.

## 1. Introduction

Maize (*Zea mays* L.) is a cereal crop that is grown worldwide and used as a staple food in some countries [1]. It is mainly used for forage, glucose, synthesizing edible oil, and other by-products [2]. Increasing heat stress around flowering is expected under climate warming [3], characterized by increased extreme high temperature events which are likely to have great negative impacts on maize production [4]. This may even result in little to no yield when severe heat stress events occur [5,6]. The influence of heat stress around flowering on ear growth and development as well as yield formation has persistently been an interesting topic for researchers in previous decades [7,8,9]. Ear length, diameter and dry matter are the basis of potential kernel number per row, kernel-row numbers, and grain yield, and are also the characteristic indexes of the growth and development dynamic of the maize ear [10]. Clarifying the effects of heating on ear growth and development dynamic is helpful to understand yield change and could provide the scientific basis for better management of maize production under global warming.

The most sensitive phases in heat stress for ear growth are ear rapid growth periods [11,12]. Heat stress during these periods easily induces a decrease in potential kernel numbers per row, kernel-row numbers, and grain yield. The rapid ear elongation period is the most critical period for determining the kernel number per ear, which is generally considered around silking (R1) [13]. Compared with ear elongation, growth rates of ear diameters and dry matter are slow and start later. The ear diameter rapidly increased from R1 to R1 + (10–14) d [12,14], and ear dry matter rapidly increased from R1 + 12 d to R1 + 38 d [11,12]. However, the current conclusions were drawn under natural field conditions, and little is known about heat stress around flowering effects on ear rapid growth periods.

Heat stress around flowering mainly includes heat stress before and after flowering, which leads to the decrease in ear morphological traits, kernel numbers, kernel weight, and grain yield [15,16,17]. Previous studies have shown that the negative effects of heat stress are larger (i) for TAS than for TBS [12,13,18], (ii) for heat-sensitive hybrid than for heat-tolerant hybrid, and (iii) for KNP than for KW [13,15]. The different effects on maize between TBS and TAS were due to their different effects on the post-silking biomass accumulation rate. Both TBS and TAS decreased the photosynthetic capacity, plant and ear dry matter [15,17,19], biomass accumulation rate and increased respiration rate [12,13,20]. Photosynthesis subjected to TBS seemed to have recovered after the treatment ended and TBS had little effect on post-silking biomass accumulation [13]. Under heat stress conditions, photosynthesis traits (e.g., chlorophyll content, photosynthetic enzyme activity, etc. [13,15]) and female and male floret characteristics (e.g., the number of tassel branches, pollen shedding amount [21], pollen viability, silk vigor, etc. [12]) led to differences in the heat tolerance of maize genotypes.

The kernel number was more sensitive to heat stress around flowering than kernel weight, and grain yield loss was mainly due to decreased kernel numbers under heat stress conditions [15,21]. TBS leads to decreased ear florets, increased ovary abortion, and decreased potential kernel numbers. Moreover, TBS decreases the number of tassel branches and tassel florets [17,22], extends the anthesis-silking interval (ASI) [6,13,23] and damages the morphology and ultrastructure of the pollen grains [17,24]. A decreased floret fertilization rate caused by pollen viability reduction is the main reason for the decrease in kernel numbers per ear under TBS [24,25]. TAS leads to a shortened duration of pollen shedding, a decreased amount of pollen shed [21,24], an inhibited endosperm cell division during the lag phase of maize and increased kernel abortion [18,26]. Kernel abortion due to an insufficient assimilate supply mainly contributes to kernel number loss under TAS [18,20]. The kernel weight is more sensitive to TAS than TBS, which may be caused by a decreased potential grain sink capacity and grain filling rate during the middle–late grain filling stage [27,28,29]. The mutual restriction between the kernel number and kernel weight as well as the source–sink flow change lead to different effects on the grain weight under heat stress around silking conditions [19,29].

In previous studies, heat stress periods mostly focused on a certain stage around flowering; however, the differences in heat stress before and after the flowering effects were not well known. The aims of this research were to (a) quantify the effects of heat stress before and after silking on the maize ear rapid growth period and growth rate; (b) determine how heat stress during different stages impacts on the yield formation of two different heat-tolerant hybrids; (c) define the relationship between ear morphological traits and yield components.

## 2. Materials and Methods

### 2.1. Crop Husbandry and Experimental Design

**Figure 1:**
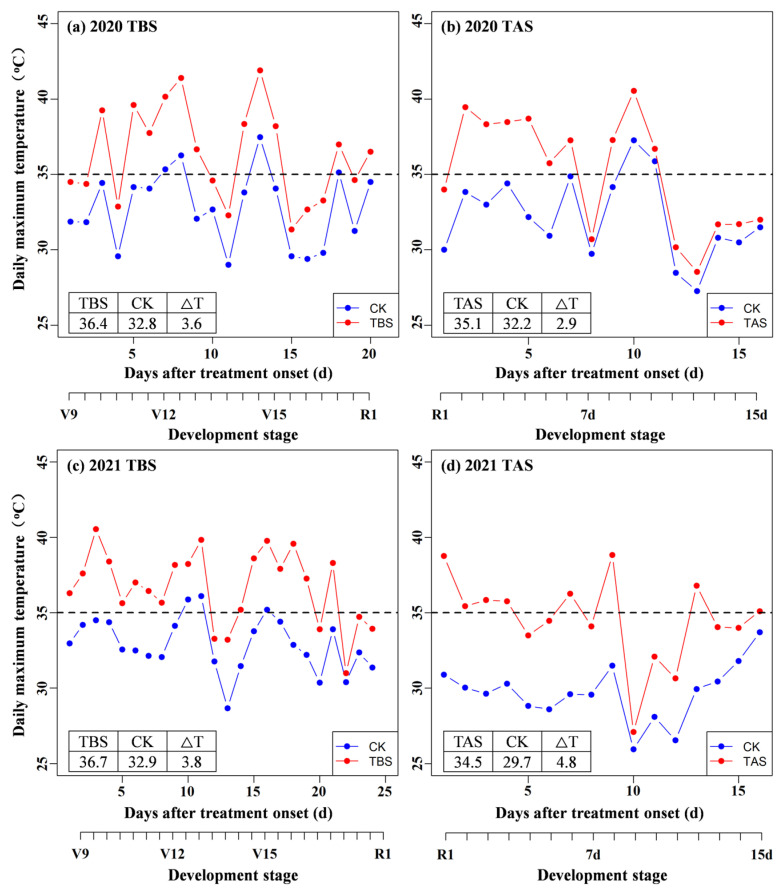
Daily maximum temperature during the key development stages under heat stress treatments in 2020 (**a**,**b**) and 2021 (**c**,**d**). TBS: Heat stress treatment before silking (V9-R1); TAS: Heat stress treatment after silking (R1-R1 + 15 d); Data in inset tables are the mean daily maximum temperature (°C) and temperature changes (ΔT, °C) under heat stress and control treatments.

Field experiments were conducted during the two successive growing seasons of 2020 and 2021. The field experiments were conducted in the experimental field of Jiyang Experimental Station (116°58′ E, 36°58′ N, altitude 21 m) on a fluvo-aquic type of soil. Treatments included a factorial combination of (i) two cultivars (a heat-tolerant maize genotype ZD958 and a heat-sensitive maize genotype DH605), (ii) three temperature regimes (CK: control with a natural field environment; TBS: heating from V9 to R1; TAS: heating from R1 to R1 + 15 d) applied during daytime hours (8:00 am to 18:00 pm, 10 h). The treatments were enclosed with polyethylene film (92% transmittance) mounted on steel structures [18]. The lateral films were opened to 60 cm above the soil surface to facilitate gas exchange. The shelters for heat stress conditions were supplemented with an automatic film winding device, climbing bar, temperature control box, circulating fan, and humidity and temperature probes (one each for the level of female and male ears). This was meant to ensure that the range of maximum daily temperature at the ear level was between 35–40 °C while the temperature in the shelters did not increase to the extent of burning the leaves. The air temperature was recorded every 10 min using HMP155 loggers (Finland VAISALA Corporation) in the shelters. All shelters were removed at the end of each heating period. The temperature increases during the maize growth period in 2020–2021 are shown in Figure 1.

Both ZD958 and DH605 cultivars were planted in each shelter (14 m long × 6 m wide × 4.5 m height, 84 m^2^ ground area), and the planting area for each cultivar was 7 m long × 6 m wide (42 m^2^ ground area). The planting density was 67,500 plant ha^−1^. The fields were sown at a 60 cm inter-row spacing and a 24 cm spacing between plants in the row. To increase the heat stress gradient around silking, cultivars were sown on three sowing dates (11 June, 19 June, 27 June). 750 kg ha^−1^ of compound fertilizer (N:P:K 15:15:15) were added to the soil in the field. At the V12 stage, 123 kg N ha^−1^ was applied as topdressing. Over the whole maize growth period, weeds, pests, and diseases were all well controlled.

### 2.2. Sampling and Measurements

#### 2.2.1. Ear Length, Ear Diameter, and Ear Dry Matter Weight

Beginning with female ear differentiation, samples of V9 (9 fully developed leaves), V12, V15, R1(silking), 7 DAS (days after silking) and 15 DAS were taken successively, after 10-day intervals until the maturity stage (R6). Three uniform plants were selected each time and the ears were husked; the ear length (from bottom to top) and ear diameter (the thickest part of the ear) were then measured. Before V12, axillary apices of maize were stripped of the ear, and the length and diameter of axillary apices were measured with a microscope scale. After V12, the ear length and diameter were measured directly with a ruler. Ears were oven-dried at 105 °C for 30 min and then at 75 °C to constant weight and weighed ear dry matter.

#### 2.2.2. Floret Differentiation and Fertilization

The uniform maize plants (three plants) in each treatment were tagged and hand-pollinated by enclosing ears in bags from silk emergence until the last emerged silks became senescent. The extra silks along the top of the husks were cut off 5–7 days after pollination. The husks were then gently peeled, and the total number of differentiated florets was counted. The counting was divided into three parts: first, after shaking gently, we counted the number of silks that were shed and the number of silks that were not shed but were wilted at the base, and added these counts to obtain the number of fertilized florets. Second, the number of fresh silks (not wilted at the base) that were not shed was taken to represent the number of unfertilized florets. Third, the number of filaments that were not drawn out of husks was taken to represent the number of degenerated florets [16]. We then calculated the floret fertilization rate and seed setting rate, using the following formula:

Floret fertilization rate (%) = number of fertilized florets/number of total florets × 100(1)

Seed setting rate (%) = kernel number/number of total florets × 100(2)

#### 2.2.3. Yield Components

At physiological maturity (R6) 35 ears were harvested from two rows (7 m long) of each plot. For each ear, ear length (EL), ear diameter (ED), ear dry matter (EDM), plant dry matter weight (PDM), kernel number per row (KNR), kernel-row number (KRN), kernel number per ear (KNP), kernel weight (KW) and grain yield (GY) were all measured. Grains per ear were dried at 75 °C to a constant weight, and then adjusted to 14% water content.

#### 2.2.4. Logistic Function and Feature Parameters

In this study, a logistic function was used to fit the growth and development dynamic of the ear length, ear diameter and ear dry matter weight and calculate the start date, end date and duration of the rapid growth period. This aimed to explore the heat stress around the flowering effects on the ear growth and development dynamic. The formulas expressing logistic function is as follows:

(3) $$y=\frac{a}{1+e^{b-ct}}$$

(4) $$t_{1}=\frac{b-1.317}{c}$$

(5) $$t_{2}=\frac{b+1.317}{c}$$

(6) $$v_{mean}=\frac{y(t_{2})-y(t_{1})}{t_{2}-t_{1}}$$

where *y* is the ear length (cm), ear diameter (cm) and ear dry matter weight (g), *t* is the number of days after ear growth (DAE, d), *a*, *b*, *c* are parameters of the function, *a* is the theoretical growth upper limit, such as the final ear length/ear diameter/ear dry matter; *t*_1_ is the beginning time of the rapid growth period, d; *t*_2_ is the end time of the rapid growth period, d; *t*_2_ − *t*_1_ is the duration of the rapid growth period, d; *v_mean_* is the mean growth rate of the rapid growth period [30]. 

### 2.3. Statistical Analyses

The mean and percentage values were calculated using Excel 2007. The treatment differences were determined by the least significant difference (LSD) and a Duncan’s test at *p* < 0.05 and a *p* < 0.01 level of probability using SPSS 17.0. MATLAB R2017a was used to fit the logistic function and draw figures. R language 4.1.0 and R package ”ggplot2” was used to produce linear fitting figures, while R package ”segmented” was used to fit the regression models with broken-line relationships.

## 3. Results

### 3.1. Effects of Heat Stress on Ear Length, Diameter, and Ear Dry Matter Weight

**Figure 2:**
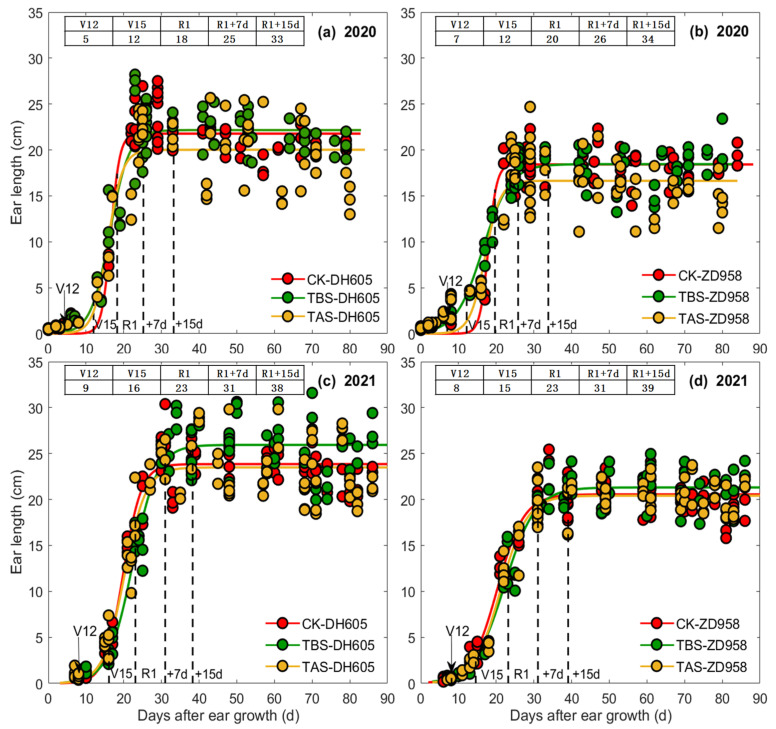
Ear lengths of DH605 (**a**,**c**) and ZD958 (**b**,**d**) at different development stages under different heat stress treatments in 2020 (**a**,**b**) and 2021 (**c**,**d**). Scatter points are all data of three sowing dates for each treatment. Inset tables show the average values of all treatments and three sowing dates. The same as in Figure 3 and Figure 4.

**Figure 3:**
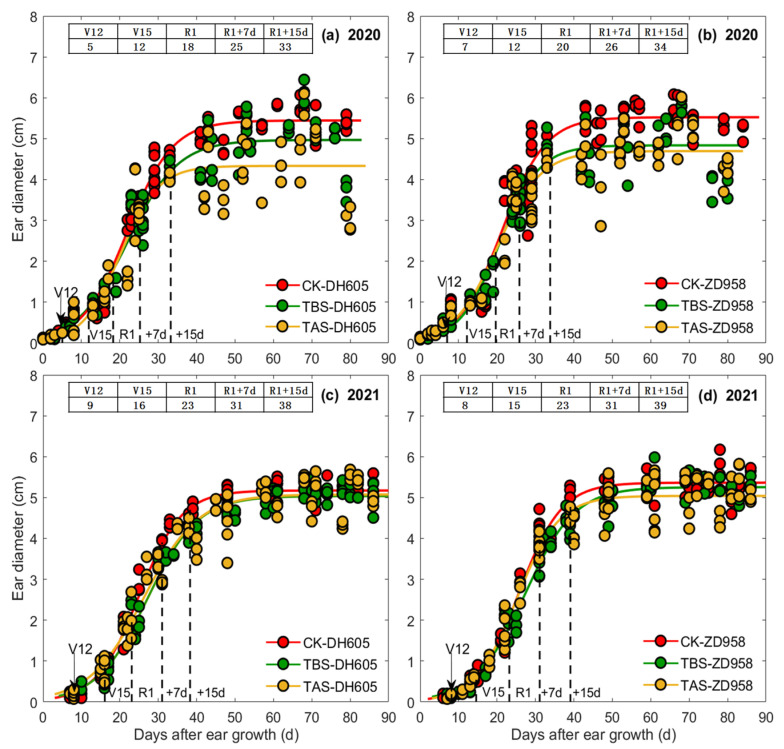
Ear diameters of DH605 (**a**,**c**) and ZD958 (**b**,**d**) at different development stages under different heat stress treatments in 2020 (**a**,**c**) and 2021 (**b**,**d**).

**Figure 4:**
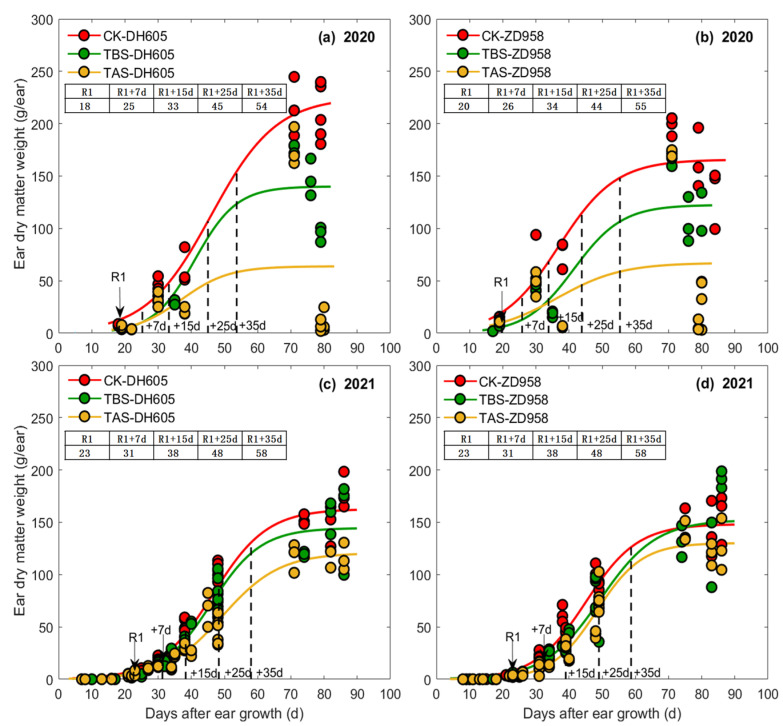
Ear dry matter weight of DH605 (**a**,**c**) and ZD958 (**b**,**d**) at different development stages under different heat stress treatments in 2020 (**a**,**b**) and 2021 (**c**,**d**).

The relationships between EL, ED, EDM and DAE were all well fitted by logistic function (Figure 2, Figure 3 and Figure 4). In general, the rapid growth periods of EL, ED and EDM of all treatments (CK, TBS and TAS) for both ZD958 and DH605 fell between V15-7 DAS, V15-15 DAS and 7–35 DAS, respectively (Figure 2, Figure 3 and Figure 4 and Table 1). The beginning time of the rapid growth period of EL/ED/EDM was almost similar among all the heat stress treatments, but the end time, duration and average grow rate during the rapid growth period were different. For EL, the end time of CK was the earliest while TBS was the last, and the duration of rapid growth period was ranked as TBS > TAS > CK. However, the average elongation rate was ranked as CK > TAS > TBS. For ED, the end time and duration of rapid growth period of ZD958 and DH605 were similar among all treatments, but the average growth rate of ED was ranked as CK > TAS > TBS. For EDM, the end time of TBS was the earliest while CK was the last, and the duration of the rapid growth period was CK > TAS > TBS. The EDM accumulation rate was ranked as CK > TBS > TAS.

Parameter *a* in the logistic equations of EL, ED and EDM of each high temperature treatment was different, indicating that the final EL, ED and EDM of ear for each treatment was different among cultivars (Figure 2, Figure 3 and Figure 4 and Table 1). For DH605, in 2020, EL (final ear length, that is, the average ear length from 15 DAS to R6, the same as below) of TBS was significantly higher than CK (6.3%) and TAS (12.9%), and EL of TAS was significantly lower than CK (5.8%). The ED (final ear diameter, that is, the average ear diameter from 25 DAS to R6, the same as below) of TBS and TAS was significantly lower than CK, which decreased by 11.8% and 21.0%, respectively, while ED of TAS was significantly lower than TBS (53.5%). In 2021, EL of TAS was significantly higher than CK (8.0%) and TBS (10.4%), and ED of TBS was significantly lower than CK (3.6%), but had no significant differences from TAS. The EDM of TBS and TAS were significantly lower than CK (14.2% and 21.4%, respectively), and EDM of TAS was significantly lower than that of TBS (18.0%). For ZD958, in 2020, EL of TBS was significantly higher than TAS (14.5%), but showed no significant difference from CK, while that of TAS was significantly lower than CK (13.3%). The ED of TBS and TAS were significantly lower than that of CK by 14.0% and 15.4%, respectively. The EDM of TBS and TAS were significantly lower than that of CK (32.9% and 64.2%, respectively), and the EDM of TAS was significantly lower than that of TBS (46.7%). In 2021, EL of TBS was significantly higher than CK (4.0%) and TAS (3.6%), and ED of TAS was significantly lower than CK (5.7%) and TBS (3.5%). There was no significant difference in ED between CK and TBS, while EDM of TBS and TAS was significantly lower than CK, with a decrease of 14.1% and 17.0%, respectively.

### 3.2. Effects of Heat Stress on Kernel Number, Kernel Weight, Yield Formation and Biomass Accumulation

TBS and TAS had no significant effects on the total number of florets in DH605 and ZD958. Compared with CK, in 2020, TBS resulted in decreased floret fertilization rates of DH605 and ZD958 by 22.2% and 21.0%, decreased KNP by 27.2% and 26.3%, decreased GY by 35.6% and 34.6%, respectively, while the seed setting rate and 1000-grain weight showed no significant change (Table 2). TBS resulted in a significant decrease in EDM of DH605 and ZD958, a significant decrease in PDM of DH605, and no significant change in the ratio of dry matter partitioning to the ear. TAS reduced the floret fertilization rate of DH605 and ZD958 by 48.2% and 35.0%, the seed setting rate by 40.1% and 38.6%, KNP by 69.3% and 50.9%, KW by 12.7% and 10.3%, and GY by 70.6% and 65.4%, respectively. TAS resulted in a significant decrease in EDM and PDM as well as the ratio of dry matter partitioning to the ear.

In 2021, TBS reduced the florets fertilization rate of DH605 and ZD958 by 17.2% and 7.8%, KNP by 22.0% and 13.2%, and GY by 19.6% and 13.9%, respectively (Table 2). TBS significantly reduced the seed setting rate of DH605 by 13.7%, while there was no significant change in the seed setting rate for ZD958 and in 1000-grain weight for DH605 and ZD958. TBS resulted in a significant decrease in PDM and EDM for DH605, and a significant decrease in EDM, but no significant change in the ratio of dry matter partitioning to the ear for ZD958. TAS decreased florets fertilization rate of DH605 and ZD958 by 18.4% and 9.5%, the seed setting rate by 14.3% and 12.2%, KNP by 23.7% and 20.4%, and GY by 27.0% and 20.3%, respectively, while KW showed no significant change or even slightly increased. TAS resulted in a significant decrease in the plant and ear dry matter weight of DH605 and ZD958, but no significant change in the ratio of dry matter partitioning to the ear.

The results of ANOVA showed that there were significant differences in the floret fertilization rate, seed setting rate, KNP, KW and EDM among years, varieties, sowing dates and heat stress treatments. GY, PDM and the ratio of dry matter partitioning to the ear were significantly different among year, sowing date and heat stress treatments. The interaction of the year and sowing date was significant for the above traits, while other interaction effects were significant for certain traits. The period and amplitude of the heat stress were the main reasons for the significant interaction between the year and sowing date.

### 3.3. Relationship between Ear Morphological Traits and Yield Components

**Figure 5:**
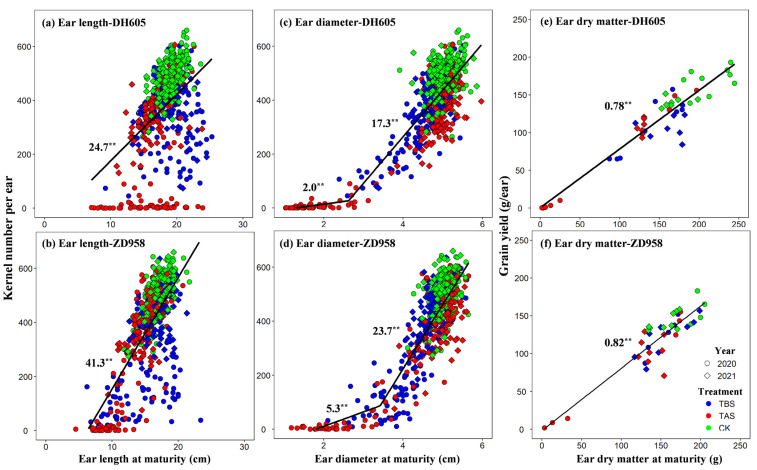
The relationship between ear length, ear diameter and kernel number per ear (**a**–**d**) and between ear dry matter and yield per ear (**e**,**f**). **, significantly different at *p* < 0.01.

EL and ED were linearly and positively correlated with KNP, and the same relationship between EDM and GY existed. Moreover, the slopes of ZD958 were greater than those of DH605 (Figure 5). Under TBS conditions, many scattered points of EL and KNP were located below the linear fit, indicating that TBS promoted the increase of EL but decreased KNP to a certain extent (Figure 5a,b). Under TAS conditions, both DH605 and ZD958 had normal EL but a smaller KNP, especially DH605, indicating that TAS seriously hindered the process of florets pollination, fertilization, and yield formation. KNP of DH605 and ZD958 increased slowly and then rapidly with ED. In the early slow increasing stage, the scatter points were all the ED and KNP under TAS, which corresponded with the scatter points with normal EL, but fewer kernels under TAS. In the rapid increasing stage, the scatter points of ED and KNP of TBS and TAS were almost on both sides of the linear fitting line, indicating that ED and KNP decreased simultaneously due to heat stress. The increase rate (kernel ratio) of KNP with EDM for DH605 and ZD958 was 0.78 and 0.82, respectively (Figure 5e,f). TAS and TBS had little effect on the kernel ratio for DH605 and ZD958.

## 4. Discussion

### 4.1. Effects of Different Levels of Heat Stress around Silking on KNP

**Figure 6:**
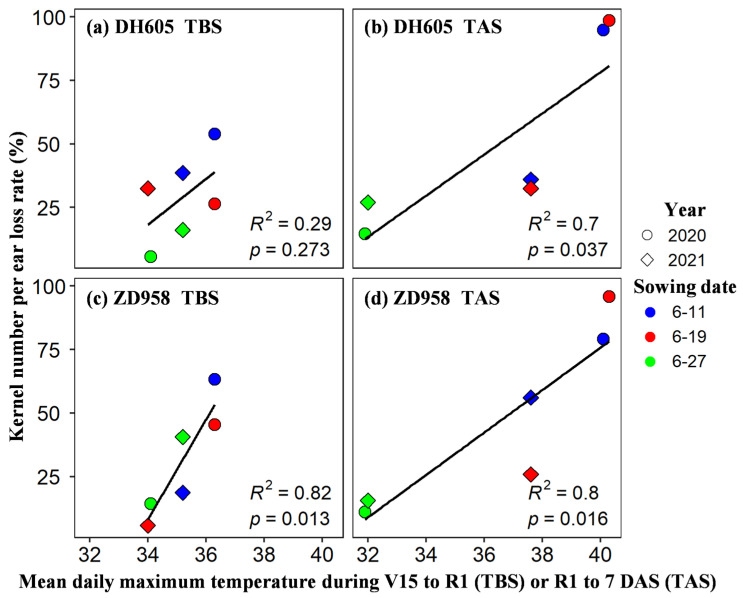
The relationship between kernel number loss rate of DH605 (**a**,**b**) and ZD958 (**c**,**d**) and mean daily maximum temperature during V15-R1 + 7 d under TBS (**a**,**c**) and TAS (**b**,**d**) conditions (TBS: V15-R1; TAS: R1-7 DAS).

The decrease in GY was mainly due to the lower KNP. In this study, the year and sowing dates, as important means to enrich heat conditions before and after silking, are crucial in comprehensively exploring the influence of different levels of heat stress around silking on the kernel number per ear. Both the year and sowing dates had significant effects on KNP, and the fundamental reason was that the higher temperature in the critical period of ear elongation (V15-7 DAS, including tasseling, silking, pollination, and fertilization) caused a higher kernel number loss rate (*p* < 0.05) (Figure 6).

### 4.2. Effects and Mechanism of Heat Stress around Silking on Ear Length, Ear Diameter and Ear Dry Matter

Heat stress before and after silking resulted in shorter, thinner, and lighter ears, which was caused by inhabited photosynthesis, enhanced transpiration, and a decreased ratio of dry matter partitioning to the ear [15,19]. However, Suwa et al. showed that the whole plant biomass was increased by heat stress, and that the stem and leaves dry matter weight increased, while EDM decreased, which further impaired hemicellulose and the cellulose synthesis of the cob [31] and decreased EL and ED. In this study, the decreased EDM mainly contributed to negative ear morphological traits (Table 2). A greater decrease in PDM and EDM under TAS caused greater influences on ear morphological traits (Table 2). Maize photosynthesis appeared to be heat-resistant. Net photosynthesis of maize was inhibited at a leaf temperature above 38 °C [32]. However, the photosynthetic rate of some maize cultivars was not reduced until 40 °C [33]. Therefore, different levels of heat stress resulted in different reasons for decreased ear morphological traits.

In this study, TBS promoted the increase in EL, which was different from the results of most previous studies. Only a few studies have drawn similar conclusions [23,31,34], but detailed reasons were not given. This study speculated that the reasons were as follows: firstly, TBS led to a decrease in the development rate before silking, a delay in the development process (about two days), a prolonged duration of the rapid increase period of EL (Table 1), and an increase in EL. Secondly, studies have shown that the elongation of the ear before silking is greatly affected by the temperature and has relatively low nutritional requirements. Appropriate warming before silking can accelerate the longitudinal division of female panicle cells, which is conducive to the increase in ear length [31]. The effect and mechanism of a high temperature on EL are still worth further exploration.

### 4.3. Effects and Mechanism of Heat Stress around Silking on Kernel Number

Three sources of kernel loss were identified: decreased floret differentiation, pollination failure, and kernel abortion. The total number of florets per ear was determined mainly by the genetic factors of cultivars, with less affection by cultivating measures than by temperature and lighting conditions around silking [35]. Edreira et al. showed that TBS caused a decrease in the number of florets among temperate and temperate × tropical hybrids [18]; however, no significant differences in the number of florets was found in this study. A decreased fertilization rate under heat stress was mainly due to decreased pollen shedding numbers, pollen shedding duration, pollen viability, silking rate, and extended ASI. The sharp decrease in the amount of pollen shedding and shortening of the duration of pollen shedding were the main reasons for the lower floret fertilization rate under TAS than TBS. Pollen aggregation, shrinking deformation and difficult anther dehiscence were the reasons for a sudden decrease in pollen shedding [23,36]. Cultivars’ responses to heat stress can be classified into female and male sensitive hybrids [21,23]. In this study, heat effects on floret fertilization rates were larger for DH605 than for ZD958, and more tassel branches and larger pollen amounts of ZD958 can alleviate the adverse effects of heat stress. Kernel abortion after fertilization was mainly attributed to inhibited endosperm cell division and insufficient assimilates supply [18,20,26].

Decreased KNR was the main reason for the decrease in KNP due to heat stress. KRN of DH605 and ZD958 decreased significantly under TAS in 2020 but not in 2021, which was caused by the higher daily maximum temperature (2.5 °C, Figure 1) and greater number of cumulative days of heat stress and heat stress intensity during the period of pollen shedding in 2020. The fertilization processes were severely hindered and KRNs were irregular. Similar conclusions were also drawn by previous studies [22].

### 4.4. Effects and Mechanism of Heat Stress around Silking on Kernel Weight

In this study, TAS decreased KW of DH605 and ZD958 in 2020, while TBS and TAS had no significant effects on KW of DH605 and ZD958, and even slightly increased, which is basically consistent with the results of previous studies [24,37]. The mutual restriction between kernel weight and kernel number per ear is an important reason for the variation in KW under heat stress conditions. Moreover, heat stress (≥35 °C) during a lag phase of maize resulted in endosperm cells division being blocked and the number of endosperm cells decreasing, then indirectly decreasing the maximum grain filling rate; and finally the KW decreased [26,29]. However, few studies have focused on the effect of the change in endosperm cell numbers on KW under heat stress around silking. The mechanism of the effect of heat stress on KW before and after anthesis is still worth further investigation.

## 5. Conclusions

Heating had negative effects on all surveyed ear and yield traits except for increased EL under TBS, and the negative effects were larger (i) for TAS than for TBS, (ii) for DH605 than for ZD958, and (iii) for KNP than for KW. A decreased growth rate during rapid ear growth periods contributed to the decreased ear traits. Floret fertilization failure and kernel abortion were the main reasons for the decrease in KNP rather than total florets. The heat stress effects on KNP were positively correlated with the daily maximum temperature during the rapid ear elongation period (V15-7 DAS). Strong linear relationships existed between ear and yield traits, which indicated that ear traits could be used as important indicators for breeding high-yield resistant varieties in the future.

## Figures and Tables

**Table 1:** Fitted parameters for the logistic equation to model ear length, ear diameter and ear dry matter growth process under different heat stress treatments. The parameters in the table were derived from all data of three sowing dates for each treatment.

Year	Cultivar	Treatment	Ear Length (EL)							Ear Diameter (ED)							Ear Dry Matter (EDM)						
			*a*	*b*	*c*	*t*_1_ (d)	*t*_2_ (d)	*t*_2_ − *t*_1_ (d)	*v_mean_* (cm d^−1^)	*a*	*b*	*c*	*t*_1_ (d)	*t*_2_ (d)	*t*_2_ − *t*_1_ (d)	*v_mean_* (cm d^−1^)	*a*	*b*	*c*	*t*_1_ (d)	*t*_2_ (d)	*t*_2_ − *t*_1_ (d)	*v_mean_* (g ear^−1^ d^−1^)
2020	DH605	TBS	22.2	6.4	0.39	13	20	7	1.9	5.0	3.4	0.16	13	29	16	0.18	140.1	6.5	0.16	32	49	17	4.9
		TAS	20.0	8.2	0.52	13	18	5	2.3	4.3	4.1	0.20	14	27	13	0.19	63.7	5.2	0.14	28	47	19	2.0
		CK	21.8	14.0	0.84	15	18	3	4.0	5.4	3.7	0.17	14	30	16	0.20	165.7	4.3	0.11	27	51	24	4.0
	ZD958	TBS	18.5	4.6	0.28	12	21	9	1.1	4.8	3.9	0.19	14	27	13	0.21	122.5	5.7	0.14	31	50	19	3.8
		TAS	16.6	8.5	0.50	14	20	6	1.6	4.7	3.5	0.18	12	27	15	0.18	67.1	3.8	0.10	25	51	26	1.5
		CK	18.4	16.0	0.89	16	19	3	3.6	5.5	3.8	0.18	14	28	14	0.23	225.2	4.7	0.10	34	60	26	4.9
2021	DH605	TBS	25.9	6.9	0.31	18	27	8	1.8	5.0	4.1	0.15	19	36	18	0.16	144.6	6.1	0.13	37	57	20	4.1
		TAS	23.5	7.2	0.35	17	24	8	1.8	5.0	4.6	0.18	18	33	15	0.20	86.6	6.5	0.14	37	56	19	2.7
		CK	23.9	8.2	0.41	17	23	6	2.1	5.2	4.5	0.18	18	32	15	0.21	148.3	5.8	0.13	34	55	21	4.2
	ZD958	TBS	21.3	5.4	0.24	17	28	11	1.1	5.2	4.2	0.15	19	37	18	0.17	152.3	5.9	0.12	38	60	22	4.0
		TAS	20.4	6.0	0.28	17	26	9	1.3	5.1	3.7	0.14	17	36	19	0.16	120.7	5.7	0.11	40	64	24	2.9
		CK	20.6	5.8	0.28	16	25	9	1.3	5.4	4.6	0.18	18	33	15	0.21	167.2	5.7	0.12	37	58	21	4.4

**Table 2:** Kernel number per ear, 1000-grain weight, yield formation process and biomass accumulation under different heat stress treatments. Values for the same treatment were the mean values under three sowing dates. Values followed by different letters within the same column for a cultivar are significantly different at *p* = 0.05. **, significantly different at *p* < 0.01, *, significantly different at *p* < 0.05, NS, the difference was not significant. The same as below.

Cultivar	Year	Treatment	Total Florets	Floret Fertilization Rate (%)	Seed Setting Rate (%)	Kernel Number Per Ear	Kernel-Row Number	Kernel Number Per Row	Kernel Number Loss Rate (%)	1000-Grain Weight (g)	Grain Yield (g)	Plant dry Matter (g)	Ear dry Matter (g)	The Ratio of Dry Matter Partitioning to the Ear
2020	DH605	TBS	752 a	68.7 b	47.4 a	370 b	16.0 a	24.8 b	27.2	309 ab	116 b	252.0 b	139.7 b	0.55 b
		TAS	756 a	42.7 c	24.9 b	156 c	11.7 b	15.8 c	69.3	289 b	53 c	214.4 b	64.9 c	0.23 c
		CK	795 a	90.9 a	65.0 a	508 a	17.2 a	31.2 a	/	331 a	180 a	339.3 a	215.0 a	0.63 a
	ZD958	TBS	581 a	69.5 b	63.8 a	365 b	14.1 ab	26.7 b	26.3	306 a	117 b	258.1 ab	121.8 b	0.46 b
		TAS	606 a	55.5 b	39.8 b	243 c	12.0 b	21.2 c	50.9	286 b	62 c	226.2 b	64.9 c	0.25 c
		CK	628 a	90.5 a	78.4 a	495 a	14.8 a	33.8 a	/	319 a	179 a	291.5 a	181.5 a	0.62 a
2021	DH605	TBS	746 a	76.7 b	48.3 b	372 b	15.5 a	26.6 b	22.0	297 a	131 b	212.3 b	140.1 b	0.66 a
		TAS	761 a	75.5 b	47.7 b	364 b	15.6 a	26.2 b	23.7	321 a	119 b	196.6 b	126.7 b	0.65 a
		CK	767 a	93.9 a	62.0 a	477 a	15.9 a	32.0 a	/	310 a	163 a	244.2 a	163.3 a	0.67 a
	ZD958	TBS	631 a	83.8 b	66.8 a	426 b	14.8 a	30.8 b	17.1	284 a	136 b	218.0 ab	133.6 b	0.61 a
		TAS	702 a	82.1 b	56.0 b	391 c	14.7 a	28.4 b	23.9	286 a	126 b	205.5 b	129.1 b	0.63 a
		CK	693 a	91.6 a	68.2 a	498 a	15.2 a	34.3 a	/	275 a	158 a	236.7 a	155.6 a	0.66 a
Source of variation	Year		**	**	**	**	**	**		**	**	**	**	**
	Cultivar		**	**	**	**	NS	NS		**	NS	NS	**	NS
	Sowing Date		*	**	**	**	**	**		**	**	**	**	**
	Treatment		**	**	**	**	**	**		**	**	**	**	**
	Year × Cultivar		**	NS	NS	**	NS	**		**	NS	NS	**	NS
	Year × Sowing date		**	**	**	**	**	**		**	**	**	**	**
	Year × Treatment		NS	**	**	**	**	**		**	**	**	**	**
	Cultivar × Sowing date		NS	NS	**	NS	NS	**		**	*	NS	NS	NS
	Cultivar × Treatment		NS	NS	*	*	NS	NS		**	NS	NS	**	NS
	Sowing date × Treatment		NS	**	**	**	NS	**		**	**	**	**	**
	Year × Cultivar × Sowing date		**	*	**	**	NS	*		**	NS	NS	NS	NS
	Year × Cultivar × Treatment		NS	NS	**	NS	NS	NS		**	NS	NS	NS	NS
	Year × Sowing date × Treatment		NS	**	**	**	**	*		**	**	**	**	**
	Cultivar × Sowing date × Treatment		NS	NS	**	*	NS	NS		**	**	*	*	NS
	Year × Cultivar × Sowing date × Treatment		*	NS	**	**	*	NS		**	**	NS	NS	NS

## Data Availability

Not applicable.
